# Three-Dimensional Structures of the Spatiotemporal Nonlinear Schrödinger Equation with Power-Law Nonlinearity in PT-Symmetric Potentials

**DOI:** 10.1371/journal.pone.0100484

**Published:** 2014-07-01

**Authors:** Chao-Qing Dai, Yan Wang

**Affiliations:** 1 School of Sciences, Zhejiang Agriculture and Forestry University, Lin'an, Zhejiang, P.R.China; 2 Optical Sciences Group, Research School of Physics and Engineering, The Australian National University, Canberra ACT, Australia; 3 Institute of Theoretical Physics, Shanxi University, Taiyuan, P.R.China; Washington State University, United States of America

## Abstract

The spatiotemporal nonlinear Schrödinger equation with power-law nonlinearity in 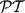-symmetric potentials is investigated, and two families of analytical three-dimensional spatiotemporal structure solutions are obtained. The stability of these solutions is tested by the linear stability analysis and the direct numerical simulation. Results indicate that solutions are stable below some thresholds for the imaginary part of 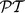-symmetric potentials in the self-focusing medium, while they are always unstable for all parameters in the self-defocusing medium. Moreover, some dynamical properties of these solutions are discussed, such as the phase switch, power and transverse power-flow density. The span of phase switch gradually enlarges with the decrease of the competing parameter *k* in 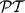-symmetric potentials. The power and power-flow density are all positive, which implies that the power flow and exchange from the gain toward the loss domains in the 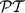 cell.

## Introduction

In the last few decades, there has been a surge of interest in obtaining exact analytical solutions of nonlinear partial differential equations (NPDEs) to describe the natural physical phenomena in numerous branches from mathematical physics, engineering sciences, chemistry to biology [1]–[3]. Exact solutions often facilitate the testing of numerical solvers as well as aiding in the stability analysis.

The nonlinear Schrödinger equation (NLSE), as one of important nonlinear models, has now become an intensely studied subjects due to its potential applications in physics, biology and other fields. Abundant mathematical solutions and physical localized structures for various NLSEs have been reported. For example, bright and dark solitons and similaritons [4]–[6], rogue waves [7], nonautonomous solitons [8] and light bullets [9] etc. have been predicted theoretically and observed experimentally in different domains.

Recently, two-dimensional accessible solitons [10] and nonautonomous solitons [11] for NLSE in parity-time (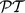)-symmetric potentials have been reported. The 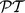-symmetry originates from quantum mechanics [12], and was introduced into optical field since the important development on the application of 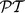
symmetry in optics was initiated by the key contributions of Christodoulides and co-workers [13]. Quite recently, various nonlinear localized structures in 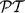-symmetric potentials have been extensively studied. Nonlinear localized modes in 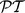-symmetric optical media with competing gain and loss were studied [14]. The dynamical behaviors of (1+1)-dimensional solitons in 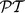-symmetric potential with competing nonlinearity were investigated [15]. Bright spatial solitons in Kerr media with 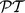-symmetric potentials have also been reported [16]. Dark solitons and vortices in 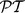-symmetric nonlinear media were discussed, too [17]. Moreover, Ruter et al. [18] and Guo et al. [19] studied the experimental realizations of such 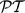 systems. However, three-dimensional (3D) spatiotemporal structures in 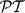-symmetric potentials are less studied. Especially, 3D spatiotemporal structures in 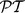-symmetric potentials with power-law nonlinearities are hardly reported.

The aim of this paper is to present 3D spatiotemporal structures of 3DNLSE with power-law nonlinearity in 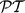-symmetric potentials. Two issues are firstly investigated in this present paper: i) analytical spatiotemporal structure solutions are firstly reported in 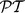-symmetric power-law nonlinear media, and ii) linear stability analysis for exact solutions and direct simulation are firstly carried out in 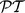-symmetric power-law nonlinear media. Our results will rich the localized structures of NLSE in the field of mathematical physics, and might also provide useful information for potential applications of synthetic 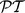-symmetric systems in nonlinear optics and condensed matter physics.

## Results

### Analytical spatiotemporal structure solutions

The propagation of spatiotemporal structures in a 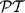-symmetric nonlinear medium of non-Kerr index is governed by the following 3DNLSE
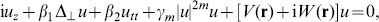
(1)where 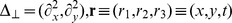, the complex envelope of the electrical field *u*(*z*,**r**) is normalized as 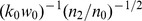 with linear index *n*_0_ and Kerr index *n*_2_, longitudinal *z*, transverse *x*,*y* coordinates and comoving time *t* are respectively scaled to the diffraction length 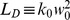, the input width unit *w*_0_ with the wavenumber 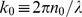 at the input wavelength *λ* and 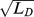. Parameters *β*_1_ and *β*_2_ are respectively the coefficients of the diffraction and dispersion, and *γ_m_* for 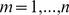 stand for the nonlinearities of orders up to 2*n*+1. For *m* = 1 one has the simple Kerr nonlinearity, for *m* = 2 the quintic, for *m* = 3 the septic, and so on. Functions 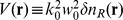 and 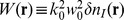, with the perturbation of index by a complex profile 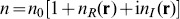, are the real and imaginary components of the complex 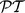-symmetric potential, and correspond to the index guiding and the gain or loss distribution of the optical potential respectively. *V* and *W* satisfy 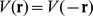 and 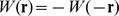.

We consider that solutions of 3DNLSE (1) is of the form:
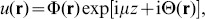
(2)where two real valued functions Φ and θ satisfy the following differential equations:
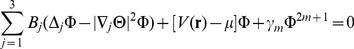
(3)

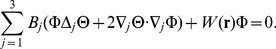
(4)where 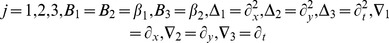.

In the following, we obtain analytical spatiotemporal structure solutions of Eqs. (3) and (4) in two different 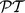-symmetric potentials.

#### Case 1 First type of extended 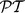-symmetric potential

Considering the 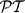-symmetric potential
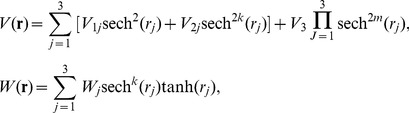
(5)with real parameters 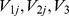 and 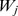, and the competing parameter *k*, the localization condition Φ→0 as **r**→±∞ yields solution of Eqs. (3) and (4)

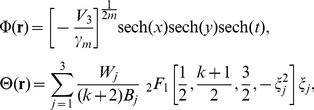
(6)where 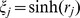 and 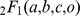 is the Hypergeometric function [20]. The parameters in the potential (4) and (5) satisfy that 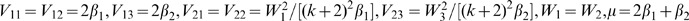, with three arbitrary constants 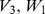 and *W*_3_. Parameter *m* has a serious impact on the nature of the gain and loss profile *W*(**r**). The value of *k* as zero or nonzero leads to *W*(**r**) as asymptotically non-vanishing or localized (asymptotically vanishing), respectively. For instance, if *k* = 0, it is the first type of extended Rosen-Morse potential, and if *k* = 1, it is the first type of extended hyperbolic Scarf potential.

From (6), 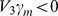, thus solution (6) exists in self-focusing (SF) media with positive nonlinearity (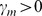) if 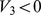, as well as in self-defocusing (SD) media with negative nonlinearity (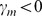) if 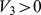.

Specially, if the value of *k* is chosen as 0–3, θ(**r**) has the different forms shown in Table 1.

**Table 1:** The expression of Θ(**r**) in first type of extended 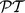-symmetric potential.

k	**Θ**(**r**)
0	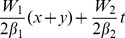
1	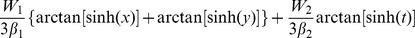
2	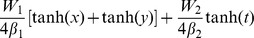
3	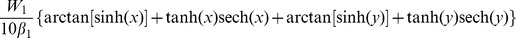 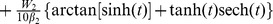

#### Case 2 Second type of extended 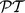-symmetric potential

In the following 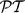-symmetric potential
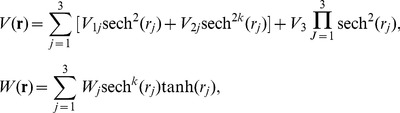
(7)with real parameters 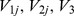 and *W_j_*, and the competing parameter *k*, the localization condition Φ→0 as **r**→±∞ leads to solution of Eqs. (3) and (4) in the form
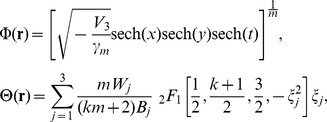
(8)with constant 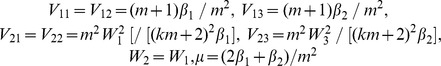
and three arbitrary constants 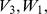 and *W*_3_. Moreover, 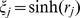 and 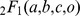 is the Hypergeometric function.

Specially, when the value of *k* is chosen as 0–3, the expressions of θ(**r**) are shown in Table 2.

**Table 2:** The expression of Θ(**r**) in second type of extended 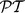 -symmetric potential.

m	Θ(r)
0	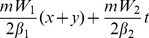
1	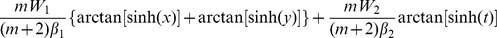
2	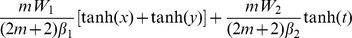
3	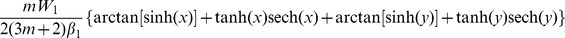 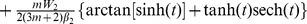

Note that for the Kerr nonlinearity (i.e. *m* = 1), solutions (6) and (8) are same.

### Properties of spatiotemporal structure solutions

**Figure 1:**
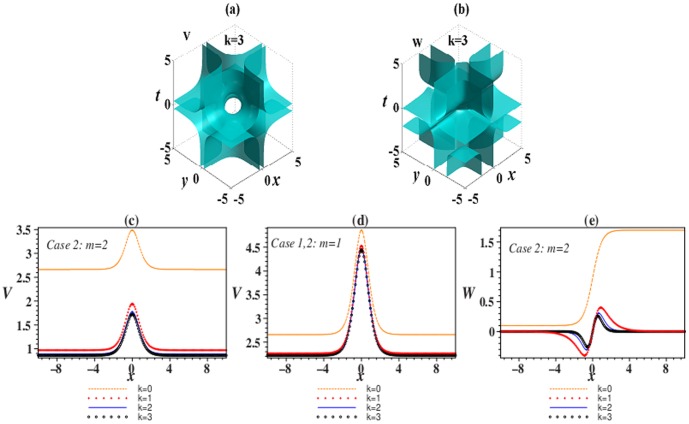
The 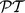-symmetric potential (7): (a) and (b) Isosurface plots of *V* and *W* for *k* = 3 at *z* = 20; (c) and (d) *V* for different *k* **at** 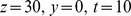 when *m* = 2 and 1, respectively; (e) *W* for different *k* at 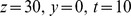 when *m* = 2. Parameters are chosen as 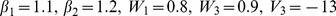.

The even and odd functions for the real part *V* and imaginary part *W* of the 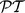-symmetric potential (7) are shown in Fig. 1 in regard to *x*,*y* and *t* for different *k*. Figs. 1(c) and (d) show *V* for different *k* at 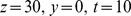 when *m* = 2 and 1, respectively. Fig. 1(e) shows *W* for different *k* at 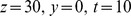 when *m* = 2. From the yellow dash lines in Figs. 1(c)–(e), *V* is localized when *m* = 1 or 2, while *W* is asymptotically non-vanishing in the 2D extended Rosen-Morse potential. It possesses unbroken 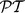-symmetry [18]. From red crosses, blue lines and black circles in Figs. 1(c)–(e), the peaks and widths of *V* and *W* gradually decrease when *k* increases. Compared red crosses, blue lines and black circles in Fig. 1(c) with those in Fig. 1(d), the amplitudes of *V* attenuate when *m* adds.

**Figure 2:**
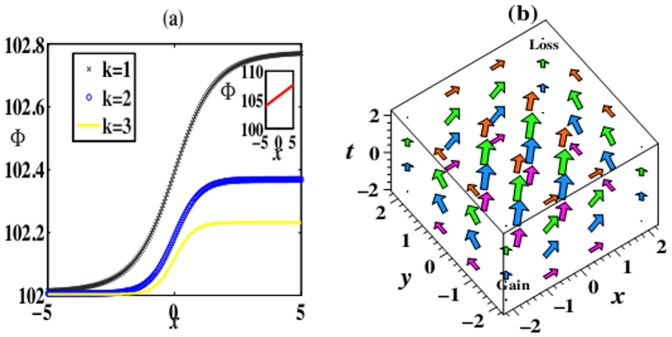
Switches of phase (8) for different 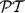-symmetric potentials (7) in (a). Power-flow vector 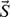 for solution (8) when *k* = 3 indicating the power flow from gain towards loss domains in (b). Parameters are chosen as the same as those in Fig. 1.

In the 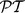-symmetric potentials above, we can find the phenomena of phase switch of solutions (6) and (8). Fig. 2(a) exhibits switches of phase in (8) for different 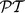-symmetric potentials (7) with *m* = 2. When *k* decreases, phases switch from smaller to bigger values along *x*, and the spans of switch gradually enlarge. However, in the Rosen-Morse potential with *k* = 0, no phase switch appears. In the 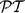-symmetric potential (5) with *m* = 1,2, the phase switch also exists. We omit the related plots.

The power *P* can be expressed as 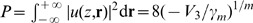 for solution (6). For solution (8), when *m* = 1, 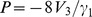, and when *m* = 2, 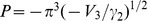. These powers are all positive for any values of the parameter *V*_3_ due to 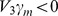, and independent of the parameter of the imaginary part in the 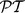-symmetric potential. Moreover, the power-flow densities (Poynting vectors) 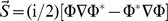 across spatiotemporal structure solutions (6) and (8) have the form 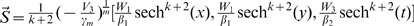 and 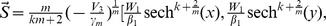 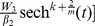, which are related to the competing parameter *k* and parameter *m*. Obviously, due to 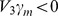, *S* is everywhere positive, which indicates that the power flow and exchange for solutions (6) and (8) in the 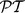 cells are always from the gain toward the loss domains (one direction). An example to this case is shown in Fig. 2(b) for *k* = 3. The similar results also exist when other *k* and *m* are chosen.

## Discussion and Analysis

### Linear stability analysis of analytical solutions

We study the linear stability of solutions (2) with (6) and (8) via the method developed in [21] when *m* = 1,2. A perturbation of an exact solution can be expressed as 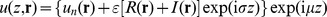, where *ε* is an infinitesimal amplitude, *u_n_*(**r**) is a solution of Eq.(1), *R*(**r**) and *I*(**r**) are the real and imaginary parts of perturbation solution, and *σ* represents the perturbation growth rate. Inserting this expression into Eq. (1) and linearizing it around the unperturbed one (the first-order term of *ε*), we have the eigenvalue problem
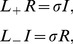
(9)where *σ* is an eigenvalue, *R* and *I* are eigenfunctions with Hermitian operators 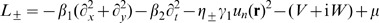 with *η*_+_ = 3 and *η*_−_ = 1 for *m* = 1 and 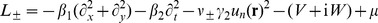 with 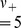 and 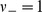 for *m* = 2. If all imaginary parts of *σ* are equal to zero, solution can be completely stable. Otherwise, if any eigenvalue *σ* possesses an imaginary part, the perturbed solution would add exponentially with *z* and thus corresponding solution becomes linearly unstable.

**Figure 3:**
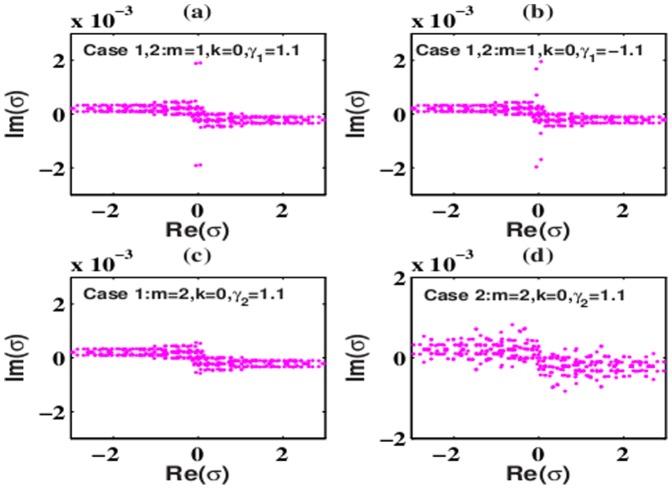
Eigenvalues for solution (6) and (8) in (a),(c),(d) SF medium and (b) SD medium under the 2D extended Rosen-Morse potential. Parameters are chosen as 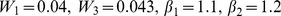 with (a),(c),(d) *V*_3_ = −13 and (b) *V*_3_ = 13. Other parameters are shown in the plots.

The eigenvalues *σ* of solutions (6) and (8) in the SF and DF media under the 2D extended Rosen-Morse potential have many imaginary parts, and thus solutions (6) and (8) are always unstable in these nonlinear media. Fig. 3 shows some examples of the eigenvalue *σ* in the SF and DF media. From Figs. 3(a) and 3(b), the eigenvalues *σ* for both SF and SD nonlinearities have many imaginary parts, and thus solutions (6) and (8) with *m* = 1 are unstable. Similar, solutions (6) and (8) with *m* = 2 are also unstable because there exist many imaginary parts of the eigenvalue *σ* in Figs. 3(c) and 3(d), too. The asymptotically non-vanishing characteristic of *W* in the 2D extended Rosen-Morse potential leads to the linear instability of solutions (6) and (8) with *m* = 1 and *m* = 2.

**Figure 4:**
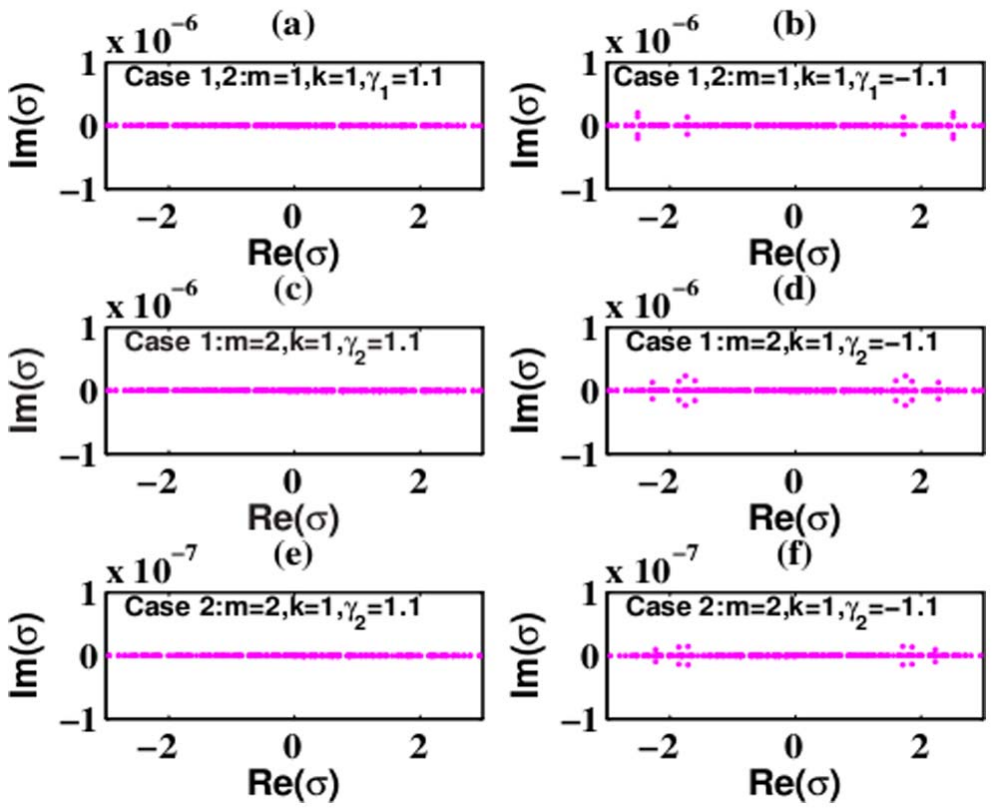
Eigenvalues for solution (6) and (8) in (a),(c),(e) SF medium and (b), (d), (f) SD medium under the 2D extended hyperbolic Scarf potential. Parameters are chosen as 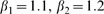 and (a),(b) Parameters are chosen as 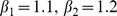 and (a),(b) 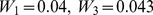, (c),(d) 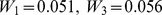, (e),(f) 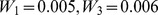 with (a),(c),(e) *V*_3_ = −13 and (b), (d), (f) *V*_3_ = 13. Other parameters are shown in the plots.

Under the 2D extended hyperbolic Scarf potential, solutions (6) and (8) with *m* = 1 and *m* = 2 are stable below some thresholds for *W*_1_ and *W*_3_ in the SF medium, while they are always unstable for all parameters in the SD medium. Fig. 4 exhibits some examples of the eigenvalues *σ* in the SF and DF media. From Figs. 4(a), (c) and (e), the eigenvalues *σ* of solutions (6) and (8) with *m* = 1 and *m* = 2 are all real, and thus solutions are linearly stable in the SF medium. When 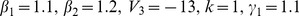 or 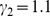, the thresholds are 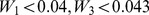 for solutions (6) and (8) with *m* = 1, 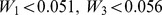 for solution (6) with *m* = 2, and 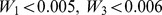 for solution (8) with *m* = 2, respectively. However, solutions (6) and (8) with *m* = 1,2 are always unstable in the SD medium because there exist some imaginary parts of the eigenvalues *σ* for all parameters. Some cases are shown in Figs. 4(b), (d), (f). From these results, the gain (loss) related to the values of 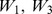 should be enough small compared with a fixed value of *V*_3_, otherwise, solutions (6) and (8) with *m* = 1 and *m* = 2 eventually lead to instability.

**Figure 5:**
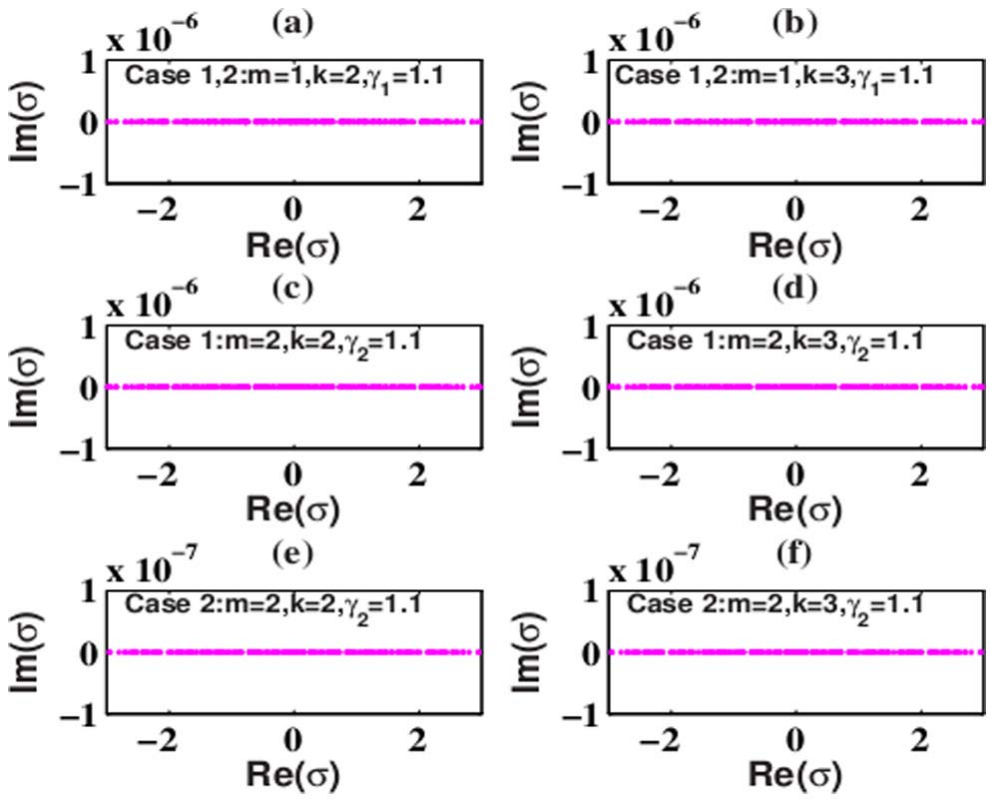
Eigenvalues for solution (6) and (8) in the SF medium under the 2D extended 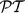-symmetric potential. Parameters are chosen as 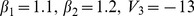 with (a) 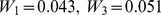, (b) 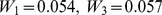, (c) 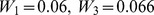, (d) 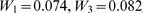, (e) 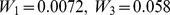 and (f) 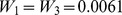. Other parameters are shown in the plots.

Furthermore, when *k* = 2,3 in the 2D extended 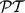-symmetric potentials (5) and (7), solutions (6) and (8) with *m* = 1 and *m* = 2 are stable below some thresholds for *W*_1_ and *W*_3_ in the SF medium because the eigenvalues *σ* of solutions (6) and (8) with *m* = 1 and *m* = 2 are all real from Fig. 3. When 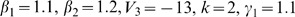 or 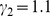, the thresholds are 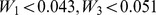 for solutions (6) and (8) with *m* = 1, 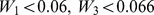 for solution (6) with *m* = 2, and 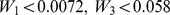 for solution (8) with *m* = 2 from Figs. 5(a), (c), (e), respectively. For 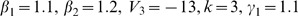 or 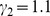, the thresholds are 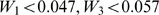 for solutions (6) and (8) with *m* = 1, 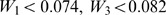 for solution (6) with *m* = 2, and 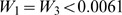 for solution (8) with *m* = 2 from Figs. 5(b), (d), (f), respectively. However, in the SD medium, solutions (6) and (8) with *m* = 1,2 are always unstable because there also exist some imaginary parts of the eigenvalues *σ* for all parameters.

When *k* is bigger, we have the similar results. Solutions (6) and (8) with *m* = 1 and *m* = 2 are stable below some thresholds for *W*_1_ and *W*_3_ in the SF medium, while they are always unstable for all parameters in the SD medium. Here we omit these discussions.

### Numerical rerun of analytical solutions

Based on the linear stability analysis, we know the stable domains of analytical solutions under different 2D extended 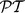-symmetric potentials. In the following, we further test the stability of these solutions by the direct numerical simulation. Here we use a split-step Fourier pulse technique. In real application, the analytical cases are not exactly satisfied, thus we consider the stability of solutions with respect to finite perturbations. The perturbations of 5% white noise are added to initial fields coming from solutions (6) and (8) of Eq. (1).

**Figure 6:**
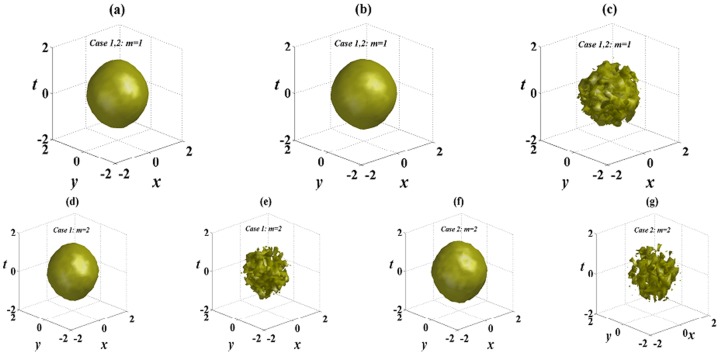
Initial value of solution (6) at *z* = 0 in (a); (b)–(g) the numerical reruns corresponding to Figs. 4(a)–(f) in the 2D extended hyperbolic Scarf potential at *z* = 80. An added 5% white noise are added to the initial values. All parameters are chosen as the same as those in Fig. 4.

Figure 6 exhibits the numerical reruns corresponding to Figs. 4(a)–(f) in the 2D extended hyperbolic Scarf potential. In the SF medium, the single 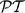 complex potential is strong enough to suppress the collapse of localized solutions caused by diffraction, dispersion and different nonlinearities. The numerical solutions in Figs. 6(b), (d) and (f) do not yield any visible instability, and good agreement with results from the linear stability analysis for analytical solutions is observed. Numerical calculations indicate no collapse, and stable propagations over tens of diffraction/dispersion lengths are observed except for some small oscillations. Compared Fig. 6(b) with Fig. 6 (d) or Fig. 6 (f) respectively, we see that for the same solution (6) or (8), solution with *m* = 1 is more stable than solution with *m* = 2 because there are smaller oscillations in Fig. 6(b) than those in Fig. 6 (d) or Fig. 6 (f). In the DF medium, solutions (6) and (8) are both unstable in the 2D extended hyperbolic Scarf potential, which is shown in Figs. 6(c), (e) and (g). They can not maintain their original shapes, change from distortion to collapse, and ultimately decay into noise.

**Figure 7:**
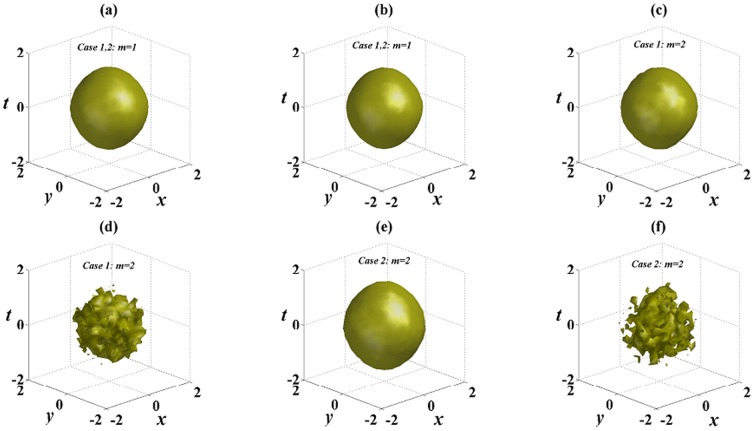
The numerical reruns corresponding to Figs. 5(a),(b),(d),(f) in the 2D extended 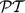-symmetric potential at *z* = 80 in (a),(b),(c),(e). (d) and (f) are corresponding to (c) and (e) in the SD medium with 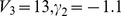. An added 5% white noise are added to the initial values. All other parameters are chosen as the same as those in Fig. 5.

Figure 7 displays other examples of stable analytical solutions, and it is the numerical reruns corresponding to Figs. 5(a),(b),(d),(f) in the 2D extended 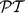-symmetric potential. In the SF medium, we can obtain stable spatiotemporal structures. From Figs. 5(a),(b),(c),(e), the influence of initial 5% white noise is suppressed, and these spatiotemporal structures (6) and (8) stably propagate over tens of diffraction/dispersion lengths and only some small oscillations appear when *k* is chosen 2 or 3 in the 2D extended 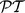-symmetric potential. However, in the DF medium, spatiotemporal structures are unstable and broken down propagating after tens of diffraction/dispersion lengths, and at last turn into noise. Compared Fig. 6(d) with Fig. 7 (c) or Fig. 6 (f) with Fig. 7 (e) respectively, spatiotemporal structures are more stable in the 2D extended 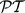-symmetric potential with *k* = 3 than those with *k* = 1.

## Conclusions

We conclude the main points offered in this paper:

- Analytical spatiotemporal structure solutions are firstly reported in 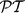-symmetric power-law nonlinear media.
  We obtain two families of analytical three-dimensional spatiotemporal structure solutions of a spatiotemporal NLSE with power-law nonlinearity in 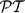-symmetric potentials. Some dynamical characteristics of these solutions are discussed, such as the phase switch, power and power-flow density. The spans of phase switch gradually enlarge with the decrease of the competing parameter *k* in 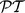-symmetric potentials. The power and power-flow density are all positive, which implies that the power flow and exchange from the gain toward the loss domain in the 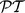
cell.
- Linear stability analysis for exact solutions and direct simulation are firstly carried out in 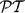-symmetric power-law nonlinear media.
  The stability of exact solutions is tested by the linear stability analysis and the direct numerical simulation. Results indicate that solutions are stable below some thresholds for the imaginary part *W* of 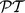-symmetric potentials in the SF medium, while they are always unstable for all parameters in the SD medium.
  Our results will rich the localized structures of NLSE in the field of mathematical physics, and might also provide useful information for potential applications of synthetic 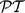-symmetric systems in nonlinear optics and condensed matter physics.
